# Is There a Role for Artificial Intelligence in the Multidisciplinary Team? A Qualitative Case-Conference Comparison in Treatment-Resistant Schizophrenia

**DOI:** 10.1192/bjo.2026.11307

**Published:** 2026-06-30

**Authors:** Lewis Kitchen, Ben Johnston

**Affiliations:** South Eastern Health and Social Care Trust, Belfast, United Kingdom

## Abstract

**Aims:**

To compare artificial intelligence (AI)-generated and consultant-generated formulation and management themes using an anonymised case of treatment-resistant schizophrenia, and to explore AI’s potential future role as a clinician-supervised adjunct in psychiatry.

**Methods:**

An anonymised longitudinal case summary of treatment-resistant schizophrenia was presented at a virtual academic meeting within the South Eastern Health and Social CareTrust, Northern Ireland, attended by approximately 50 clinicians including 32 consultant psychiatrists. No patient-identifiable information was used, shared, or processed in the case materials or subsequent write-up. Consultant priorities were captured and synthesised by the case presenter into core themes.

In parallel, the same anonymised case narrative was discussed within a single AI conversation using an OpenAI large language model (GPT-5.2 Thinking). The AI-generated themes were informed by the case narrative and an uploaded specialist prescribing reference (Maudsley Prescribing Guidelines, 15th edition). Themes were compared for overlap and difference with reference to evidence-aligned core domains relevant to treatment-resistant schizophrenia.

**Results:**

The consultant group focused on the clozapine pathway, including whether clozapine had received an adequate trial and whether a monitored re-challenge could be considered. Discussion revisited reasons for discontinuation and relevant physical parameters, including electrocardiogram findings and inflammatory and cardiac blood markers. A second theme was improving treatment delivery through adherence strategies, including long-acting injectable antipsychotics, particularly haloperidol depot based on prior tolerability. The consultant group also highlighted tertiary specialist input for complex clozapine risk–benefit decisions amid substance misuse, risk, and psychosocial complexity; this was not proposed by the AI output. Electroconvulsive therapy was not openly proposed by the consultant group but was identified by the AI output as a potential consideration. Augmentation options, such as allopurinol, were noted by both groups.

Overall, the AI output proposed a similar pattern of thinking across major priorities and added value through structure and sequencing.

**Conclusion:**

The consultant group and AI-generated themes were broadly similar andreflected guideline-consistent common practice in treatment-resistant schizophrenia, with differences in emphasis and service-level options. The consultant group contributed a grounded sense of feasibility shaped by engagement patterns, service capacity, and psychosocial circumstances. AI’s benefit was structured presentation, with coherent formulation domains and sequencing of options and monitoring considerations.

This supports a role for AI as a clinician-supervised adjunct for formulation, treatment planning, documentation support, and education, without replacing clinician judgement or accountability. Future studies should plan the comparison in advance, use more than one independent rater, and test whether artificial intelligence support improves process measures under appropriate information governance.

